# From anti-inflammation to pro-resolution: a new paradigm for specialized pro-resolving mediators in regulating neuroinflammation and repair after cerebral ischemia-reperfusion

**DOI:** 10.3389/fimmu.2026.1709410

**Published:** 2026-05-08

**Authors:** Xinna Wang, Hang Chen, Yan Xu, Hongtao Cui, Fang Liu

**Affiliations:** 1 Department of Encephalopathy, Affiliated Hospital of Changchun University of Chinese Medicine, Changchun, China; 2 Department of Pediatrics, The First Affiliated Hospital, Henan University of Chinese Medicine, Zhengzhou, Henan, China; 3 School of Pediatrics, Henan University of Chinese Medicine, Zhengzhou, Henan, China; 4 Department of Pediatrics, Chongqing Traditional Chinese Medicine Hospital, Chongqing, China; 5 The First Affiliated Hospital of Chongqing University of Chinese Medicine, Chongqing, China; 6 Department of Endocrinology and Metabolism, Chongqing Traditional Chinese Medicine Hospital, Chongqing, China

**Keywords:** anti-inflammation, cerebral ischemia-reperfusion, inflammation to pro-resolution, neuroinflammation, specialized pro-resolving mediators

## Abstract

Uncontrolled neuroinflammation following cerebral ischemia-reperfusion is a core pathophysiological process driving secondary brain injury and leading to long-term neurological dysfunction. For decades, traditional “anti-inflammatory” strategies targeting the inhibition of key pro-inflammatory pathways have repeatedly failed in clinical translation, compelling a fundamental re-evaluation of the biological nature of inflammation. Inflammation is not a process that passively subsides upon stimulus removal but a dynamic one that requires active “resolution” through endogenous programs to restore tissue homeostasis. Within this precisely regulated program, a family of endogenous lipid mediators derived from polyunsaturated fatty acids—Specialized Pro-resolving Mediators (SPMs)—act as central executors. This review systematically proposes a translational framework for post-stroke inflammation management, shifting from traditional “passive anti-inflammation” to “active pro-resolution.” We first delve into the translational challenges and theoretical limitations of conventional anti-inflammatory therapies. Subsequently, we elaborate on the biosynthetic network of SPMs, their major families (lipoxins, resolvins, protectins, and maresins), and their pleiotropic biological functions, including halting neutrophil infiltration, reprogramming macrophage/microglial functions, enhancing the efficiency of apoptotic cell clearance (efferocytosis), and maintaining blood-brain barrier integrity. The central thesis of this review is that a key mechanism underlying the persistent neuropathological deterioration after cerebral ischemia-reperfusion is the failure of the endogenous inflammation resolution program, termed “resolution dysfunction.” By integrating mounting clinical evidence with extensive preclinical studies, this review provides a systematic argument for this hypothesis. Exogenous administration of SPMs or their stable analogs has demonstrated significant neuroprotective effects in various animal models, effectively reducing infarct volume and improving functional outcomes. Finally, this review explores the critical challenges in developing SPMs into novel stroke therapies, such as pharmacokinetics, therapeutic windows, and targeted delivery, and poses forward-looking questions for future research in the field. In summary, targeting and restoring the brain’s endogenous inflammation resolution programs not only offers a promising new strategy for stroke treatment but also represents a profound practice of a disruptive therapeutic philosophy aimed at promoting the restoration of tissue homeostasis.

## 1 Introduction

Ischemic stroke, a major cerebrovascular event, imposes a heavy socioeconomic burden globally due to its high incidence, disability, and mortality rates (1, 2). In the acute phase, rapid restoration of blood flow to the occluded vessel via intravenous thrombolysis (rt-PA) or mechanical thrombectomy is the cornerstone of modern stroke therapy. The primary goal is to salvage neurons within the ischemic penumbra, which are in a state of reversible injury (3). However, clinical practice shows that a significant proportion of patients have poor neurological outcomes even after successful revascularization. The key pathophysiological process behind this phenomenon is “Ischemia-Reperfusion Injury” (IRI). One of the core features of IRI is a severe and often uncontrolled neuroinflammatory response, accompanied by an explosive burst of oxidative stress and activation of apoptotic programs (4). Although inflammation plays a crucial physiological role in the early stages by clearing cellular debris from primary ischemic necrosis, such as the phagocytic removal of dead neurons by microglia (5), its excessive and sustained activation turns it into the other edge of a “double-edged sword.” A persistent inflammatory storm exacerbates the disruption of the Blood-Brain Barrier (BBB), induces secondary death of neurons and glial cells, and ultimately inhibits endogenous neurorestorative and angiogenic processes, thereby exerting a profound negative impact on the patient’s long-term neurological prognosis (6).

Based on the recognition of neuroinflammation’s destructive potential, drug development over the past decades has largely focused on “anti-inflammatory” strategies. Researchers have attempted to intervene at multiple points in the inflammatory cascade, ranging from targeting leukocyte adhesion with an anti-Intercellular Adhesion Molecule-1 (ICAM-1) antibody (Enlimomab) (7, 8), neutralizing key pro-inflammatory cytokines like Tumor Necrosis Factor-α (TNF-α) with drugs such as Etanercept (9), and scavenging oxidative stress products with free-radical scavengers like NXY-059 (10). Additionally, antagonists targeting the interleukin-1 receptor (Anakinra) have also undergone clinical trials (11). However, a thought-provoking phenomenon is that while these strategies generally showed significant neuroprotective effects in preclinical animal models, they almost invariably failed to meet the primary endpoint of improving long-term functional outcomes in large-scale, randomized controlled human clinical trials, and in some cases, even showed harmful effects (12). This persistent bench-to-bedside translational dilemma forces a fundamental reassessment of the traditional view that treats inflammation merely as an “enemy” to be completely and non-selectively suppressed. The inherent flaw in this strategy may be that it ignores the complexity and duality of the inflammatory response; while suppressing its destructive effects, it may also interfere with its beneficial functions in clearing tissue debris, promoting angiogenesis, and initiating tissue repair (4).

This series of translational failures points to a fundamental issue: viewing inflammation as a purely destructive process that only needs to be suppressed is likely an oversimplified perspective. Groundbreaking research has revealed that the successful termination of inflammation is not a passive decay process but an active, highly coordinated biological program mediated by endogenous signaling molecules, known as the “Resolution of Inflammation” (13, 14). This program involves a series of ordered events, including the active inhibition of further neutrophil infiltration, induction of their apoptosis, and promotion of their efficient clearance by macrophages (i.e., efferocytosis). The ultimate goal is to actively “shut down” the inflammatory response after effectively clearing the injury or pathogen, thereby minimizing tissue damage and initiating repair to restore tissue homeostasis (13, 14). In this sophisticated regulatory network, a family of lipid mediators derived from polyunsaturated fatty acids (PUFAs)—Specialized Pro-resolving Mediators (SPMs)—plays the key role of “master conductors” (15, 16). These molecules, primarily comprising four major families—Lipoxins (LXs), Resolvins (Rvs), Protectins (PDs), and Maresins (MaRs)—exert potent biological activities at very low concentrations. By binding to specific receptors, they precisely regulate immune cell function, guiding the transition of inflammation from a destructive phase to a reparative one (16, 17).

Against this backdrop, This review proposes a translational framework for ischemic stroke, applying the well-established pro-resolution biology—pioneered by Serhan and colleagues over two decades ago—to a clinical field that has been dominated by suppression-based anti-inflammatory strategies (18). While the pro-resolution paradigm is not new, its systematic application as a therapeutic philosophy for stroke remains underexplored. This paper will systematically elucidate the biological basis of the inflammation resolution program and delve into the biosynthesis, functions, and critical role of SPMs in cerebral ischemia-reperfusion injury. By integrating the latest evidence from biochemistry, cell biology, preclinical models, and clinical observations, the core purpose of this review is to systematically argue for a key scientific hypothesis: a core pathophysiological link in the persistent neurological deterioration after cerebral ischemia-reperfusion is the dysfunction or failure of the endogenous inflammation resolution program. This perspective—restoring and enhancing the brain’s own capacity for inflammation resolution and tissue repair by exogenously supplementing SPMs or boosting their endogenous biosynthesis aimed at restoring tissue homeostasis, opening a hopeful new path for the development of next-generation stroke therapies.

## 2 The double-edged sword of post-stroke inflammation: temporal dynamics from initiation to resolution

Neuroinflammation following ischemia-reperfusion is not a single, static event but a complex process that evolves dynamically in both time and space. From a biological function standpoint, this process can be clearly divided into three continuous yet intertwined phases: the “initiation” phase triggered by damage signals, the “amplification” phase characterized by massive immune cell infiltration, and the “resolution” phase ultimately aimed at restoring tissue homeostasis (19). Understanding the key cellular and molecular events of each phase is crucial for identifying potential therapeutic targets.

### 2.1 The acute pro-inflammatory storm

Within minutes to hours after the reperfusion event, dying or necrotic neurons and glial cells in the ischemic region release large quantities of endogenous danger signals, known as Damage-Associated Molecular Patterns (DAMPs). These molecules act as “alarmins,” initiating a sterile inflammatory response. Among them, High-Mobility Group Box 1 (HMGB1) released from the nucleus (20), adenosine triphosphate (ATP) leaked from the cytoplasm (21), S100B protein (22), and mitochondrial DNA (mtDNA) (23) and formyl peptides derived from damaged mitochondria (24) are the most extensively studied DAMPs. The release of these DAMPs is not only a consequence of cell death but also has active signaling functions; for instance, HMGB1 can be actively secreted by activated immune cells, further amplifying the inflammatory signal.

These DAMP molecules are rapidly recognized by the brain’s resident immune cells—primarily microglia and astrocytes—through their cell surface or cytoplasmic Pattern Recognition Receptors (PRRs) (25). Toll-like receptors (TLRs), particularly TLR2 and TLR4, are key receptors for recognizing various DAMPs. The binding of DAMPs to TLRs primarily activates the Myeloid differentiation primary response 88 (MyD88)-dependent signaling pathway, leading to the phosphorylation of downstream kinases (such as IRAKs) and activation of TRAF6. This ultimately releases the inhibition of the nuclear factor-κB (NF-κB) inhibitor protein IκB, promoting NF-κB activation and nuclear translocation (26, 27). As a key pro-inflammatory transcription factor, nuclear NF-κB initiates the explosive transcription of numerous pro-inflammatory genes, including various cytokines like TNF-α, Interleukin-1β (IL-1β), and Interleukin-6 (IL-6), as well as chemokines (28). Critically, lipid mediators are among the earliest biochemical responders in the acute inflammatory cascade, preceding cytokine amplification. In sterile injury such as ischemia-reperfusion, membrane phospholipid-derived prostaglandin E_2_(PGE_2_), generated via cyclooxygenase (COX)-mediated oxidation of arachidonic acid, acts within minutes to induce vasodilation, increase vascular permeability, and sensitize nociceptors (29). In parallel, leukotriene B_4_(LTB_4_), produced via the 5-lipoxygenase pathway, is a potent chemoattractant driving early neutrophil recruitment (30). The clinical efficacy of NSAIDs—which act by inhibiting COX-1 and COX-2, thereby blocking PGE_2_ production— directly validates the central role of these early lipid mediators in driving acute inflammation. However, as discussed in Section 1, the same COX enzymes, when acetylated by aspirin, also contribute to the biosynthesis of pro-resolving SPMs, underscoring the double-edged nature of targeting this pathway non-selectively.

In the production of pro-inflammatory cytokines, the activation of the NLRP3 inflammasome plays a crucial role. The activation of the NLRP3 inflammasome typically follows a “two-signal model”: Signal one (priming), usually provided by NF-κB activated via the TLR pathway, leads to the transcriptional upregulation of NLRP3 and pro-IL-1β; Signal two (activation) can be provided by various stimuli, such as potassium efflux mediated by extracellular ATP via the P2X7 receptor, production of mitochondrial Reactive Oxygen Species (ROS), or lysosomal rupture (31). The combined action of these two signals leads to the oligomerization of the NLRP3 protein, which then recruits the apoptosis-associated speck-like protein containing a CARD (ASC) and pro-caspase-1 to assemble a functional inflammasome complex. On this complex, pro-caspase-1 is cleaved and activated into caspase-1, which in turn cleaves pro-IL-1β and pro-IL-18, leading to their maturation and release, thereby greatly amplifying the inflammatory response (31, 32).

The early-produced inflammatory mediators rapidly disrupt the structural and functional integrity of the BBB, opening the way for peripheral immune cell infiltration (33). Guided by chemokines, neutrophils are the first circulating leukocytes to arrive at the injury site. They roll, adhere to, and transmigrate across the activated cerebrovascular endothelium via selectins and integrins on their cell surface (34). Infiltrated neutrophils exacerbate oxidative stress and tissue damage by releasing a variety of weapons, including ROS, Myeloperoxidase (MPO), and neutrophil elastase. A particularly critical pathological process is the formation of Neutrophil Extracellular Traps (NETs), a process known as NETosis. This involves the decondensation of neutrophil chromatin and rupture of the cell membrane, releasing a web-like structure composed of a DNA fiber backbone, histones (e.g., H3, H4), and granular proteins (e.g., elastase, MPO). These NETs not only cause a “no-reflow” phenomenon by physically obstructing microvessels but their components (especially histones) are also potent DAMPs and platelet activators, further amplifying inflammation and thrombosis (35, 36). Subsequently, a large number of monocyte-macrophages also infiltrate and primarily differentiate into the pro-inflammatory M1 phenotype (induced by signals like IFN-γ and LPS, with high expression of iNOS and CD86), further amplifying the inflammatory storm (37, 38).

### 2.2 The inflection point of inflammation: switching from pro-inflammation to pro-resolution

A functional inflammatory response must be effectively terminated after completing its initial task to avoid excessive damage to the host tissue. Within days after a stroke, the inflammatory microenvironment begins to undergo a critical transformation. A large number of early-infiltrating neutrophils initiate apoptosis and, through a complex series of molecular signals, guide phagocytes to clear them. This process, known as “efferocytosis” (from the Greek, meaning “to carry the dead”), is the central turning point for inflammation to shift from a destructive to a reparative state (13, 14). Apoptotic cells actively release “find-me” signals, such as lysophosphatidylcholine (LPC), sphingosine-1-phosphate (S1P), and the chemokine CX3CL1, to recruit phagocytes from a distance (39, 40). As phagocytes approach, “eat-me” signals exposed on the outer membrane of apoptotic cells, most typically phosphatidylserine (PS), are recognized by various receptors on the phagocyte surface (e.g., TIM-4, BAI1) or indirectly via bridging molecules (e.g., Gas6, Protein S) by phagocytic receptors (e.g., MerTK, AXL) (41, 42).

Efferocytosis is far more than a simple “garbage disposal” process; it is a powerful immunomodulatory signaling event. After engulfing apoptotic cells, the internal signaling pathways of phagocytes are actively reprogrammed. For example, upon binding to an apoptotic cell, the MerTK receptor activates the downstream Rac1 signal, promoting phagosome formation (43). Simultaneously, it inhibits the pro-inflammatory NF-κB pathway and activates anti-inflammatory and pro-reparative signaling pathways (such as STAT3/STAT6). This causes phagocytes (mainly microglia and macrophages) to switch from a pro-inflammatory “combat state” to an anti-inflammatory and pro-reparative “stabilizing state.” This is manifested by the cessation of secreting pro-inflammatory cytokines like TNF-α and IL-1β, and instead, the massive secretion of potent anti-inflammatory mediators such as Interleukin-10 (IL-10) and transforming growth factor-β (TGF-β) (44).

Parallel to this process, under the influence of specific cytokines like IL-4 and IL-13, the phenotype of macrophages/microglia also begins to shift from the classic pro-inflammatory M1 phenotype to the pro-reparative M2 phenotype. However, it must be emphasized that the M1/M2 model is a highly simplified binary concept derived from *in vitro* studies. In the complex *in vivo* pathological microenvironment, the activation state of microglia should be viewed as a continuous functional spectrum (45). In recent years, high-dimensional analysis techniques such as single-cell transcriptomics have revealed more functionally specific cell subpopulations, such as “Disease-Associated Microglia” (DAMs) found in various neurodegenerative diseases. These cells often co-express classic M1 and M2 markers and have unique gene expression profiles (46). How SPMs finely regulate these more complex cell subpopulation states is a frontier of current research. Nevertheless, the “tendency shift from M1 to M2” remains a valid conceptual framework for describing the transition of the inflammatory microenvironment from destructive to reparative.

**Figure 1:**
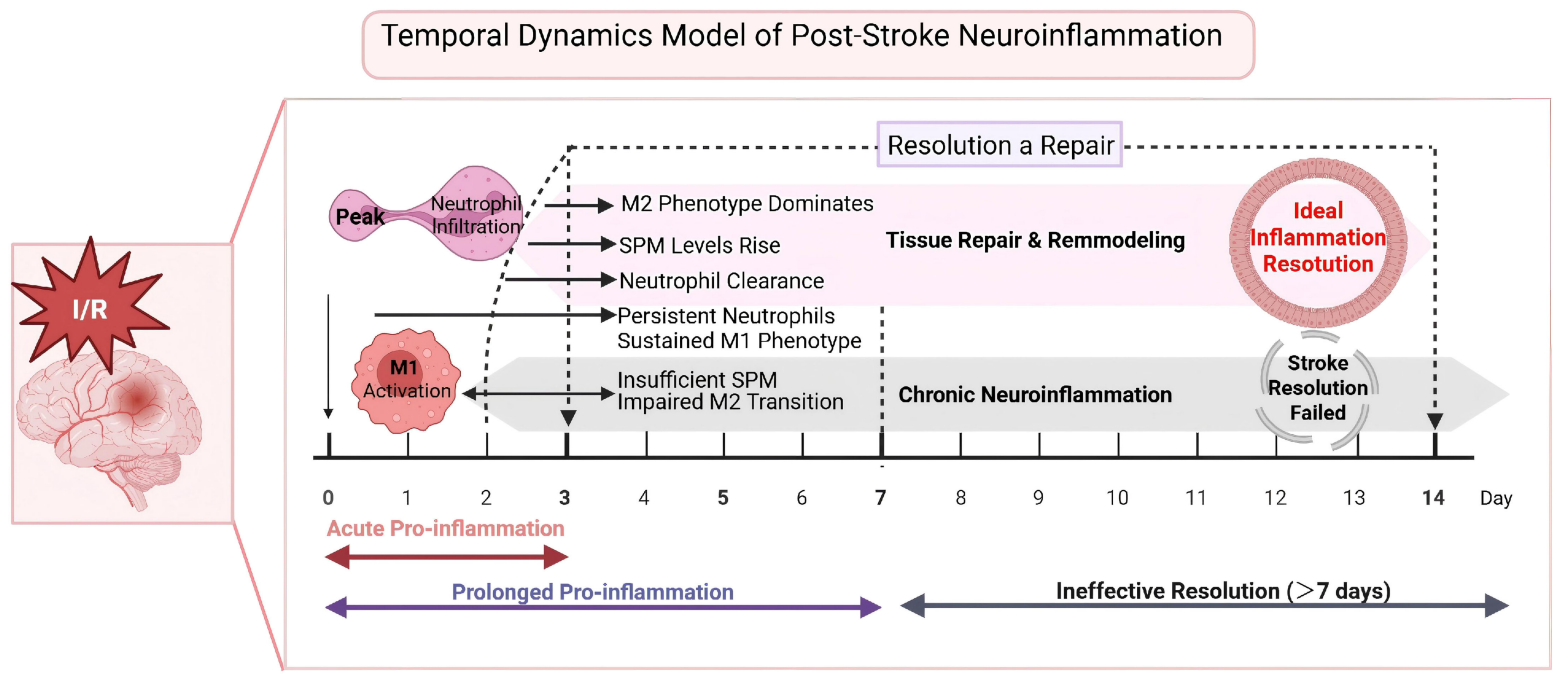
This diagram contrasts the timeline of an ideal inflammation resolution process (top) with the common “failed resolution” scenario after stroke (bottom). In an ideal situation, the pro-inflammatory phase is significantly prolonged, and the resolution program fails to initiate—a state we term ‘non-resolving neuroinflammation’. We acknowledge that the relationship between acute and chronic inflammation is not a simple linear continuum; the two processes involve distinct biological mechanisms. The pathological state described here is more precisely characterized as failed resolution of the acute inflammatory response, which creates conditions permissive for sustained tissue damage, rather than a direct transition into classical chronic inflammation.

Crucially, within the same time window as the cellular functional shift, a key “Lipid Mediator Class Switching” occurs in the intracellular lipid mediator biosynthesis pathways (18). The synthesis pathways switch from preferentially producing potent pro-inflammatory mediators, such as Prostaglandin E2 (PGE_2_) and Leukotriene B4 (LTB_4_), to preferentially synthesizing pro-resolving SPMs (14, 47). This biochemical shift forms the molecular basis for the transition of inflammation from amplification to resolution, paving the way for subsequent tissue repair and functional recovery (**Figure 1**).

## 3 Specialized pro-resolving mediators: the “master conductors” of inflammation resolution

Unlike traditional anti-inflammatory drugs, which primarily act through inhibition, SPMs are not simple “inhibitors” of inflammation but active “coordinators” of its resolution. These endogenous lipid mediators exert powerful and highly specific biological activities at very low physiological concentrations (typically in the nanomolar to picomolar range) (48–50). This chapter will detail the major families of SPMs, their complex biosynthetic networks, and the pleiotropic biological functions they perform in regulating inflammation resolution and tissue repair.

### 3.1 Families and biosynthesis of SPMs

The biosynthesis of SPMs begins with the release of PUFAs from cell membrane phospholipids by phospholipase A2 (PLA2). These PUFAs mainly include the omega-6 fatty acid arachidonic acid (AA) and the omega-3 fatty acids eicosapentaenoic acid (EPA) and docosahexaenoic acid (DHA) (51). These PUFAs then undergo a series of highly stereoselective oxidation reactions catalyzed by members of the lipoxygenase (LOX) and cyclooxygenase (COX) families to produce biologically active SPM molecules. This process often requires close collaboration between different cell types, known as transcellular biosynthesis (52).

Lipoxins (LXs) are the first SPM family to be discovered and are primarily derived from AA. One of their classic synthesis pathways involves the 5-LOX-catalyzed generation of the unstable epoxide intermediate leukotriene A4 (LTA_4_) from AA in one cell (e.g., a neutrophil). LTA_4_ is then transferred to an adjacent cell (e.g., a platelet), where it is metabolized by 12-LOX to produce LXA_4_ and LXB_4_ (53, 54).

Resolvins (Rvs) are an SPM family derived from omega-3 PUFAs, divided into the E-series and D-series based on their precursors. E-series resolvins (e.g., RvE1, RvE2) originate from EPA. Under specific conditions (such as the presence of aspirin), acetylated COX-2 can catalyze EPA to form 18R-hydroxy-eicosapentaenoic acid (18R-HEPE), which is then acted upon by 5-LOX to generate RvE1 and RvE2 (49, 55). D-series resolvins (e.g., RvD1-RvD6) are derived from DHA. In human cells, DHA is first catalyzed by 15-LOX-1 (12/15-LOX in mice) to form 17S-hydroperoxy-DHA (17S-HpDHA). This unstable intermediate can be further metabolized by 5-LOX through epoxidation and hydrolysis steps to generate various D-series members, including RvD1 and RvD2 (56).

Protectins (PDs) and Maresins (MaRs) are both derived from DHA. The synthesis of protectins (e.g., PD1, also known as NPD1 in the nervous system) is also initiated by 15-LOX, but its subsequent enzymatic reaction pathway differs from that of resolvins, ultimately forming a molecule with a conjugated triene structure (57). The synthesis of maresins (e.g., MaR1, MaR2) is cell-specific, primarily catalyzed by 12-LOX in macrophages, and their name highlights their important role in regulating macrophage function (17).

Beyond the major SPM families derived from AA, EPA, and DHA, other SPMs have been identified from alternative precursors. Notably, n-3 docosapentaenoic acid (n-3 DPA) serves as a precursor for n-3 DPA-derived resolvins, protectins, and maresins, as well as for 13-series resolvins (also called T-series resolvins). Aspirin-triggered (AT) isoforms of SPMs, including AT-lipoxins, AT-resolvins, and AT-protectins, are generated via aspirin-acetylated COX-2 (37, 38).

### 3.2 Pleiotropic biological functions of SPMs

SPMs initiate downstream signaling pathways by binding to specific G protein-coupled receptors (GPCRs) on the cell surface, thereby exerting their broad and precise regulatory effects. These receptors include ALX/FPR2 (recognized by both LXA_4_ and RvD1), GPR32 (another receptor for RvD1), ChemR23 (receptor for RvE1), and GPR18 (receptor for RvD2). Their pleiotropic biological functions are mainly reflected in the fine-tuning of the immune response and the direct protection of tissue homeostasis.

In terms of immune regulation, SPMs first “apply the brakes” to inflammation by halting neutrophil infiltration. This is the critical first step in initiating inflammation resolution. For example, LXA_4_, acting on the ALX/FPR2 receptor on the neutrophil surface, and RvE1, acting on the ChemR23 receptor, collaboratively inhibit downstream signals induced by chemoattractant lipid mediators like LTB_4_ (58). This blocks the rearrangement of the actin cytoskeleton, thereby inhibiting chemotaxis and transendothelial migration. Second, SPMs are potent enhancers of phagocytosis, efficiently promoting the clearance of apoptotic cells and cellular debris. RvD1, through its receptors GPR32 and ALX/FPR2 on human cells, significantly upregulates phagocytic receptors on macrophages (e.g., TIM-4, MerTK) responsible for recognizing apoptotic cells and activates downstream signaling molecules like Rac1, thereby greatly increasing the efficiency of efferocytosis (59). Third, SPMs actively reprogram the function of immune cells, shifting them from a pro-inflammatory to an anti-inflammatory and pro-reparative phenotype. For instance, molecules like RvD1 and MaR1 can effectively “push” microglia/macrophages from a pro-inflammatory M1 phenotype to a pro-reparative M2 phenotype by activating PPAR-γ signaling or inhibiting the phosphorylation of STAT1, shifting them from pro-inflammatory M1 to pro-reparative M2 phenotypes. This transforms them from “amplifiers” of inflammation to “repairers” of tissue (60, 61).

**Figure 2:**
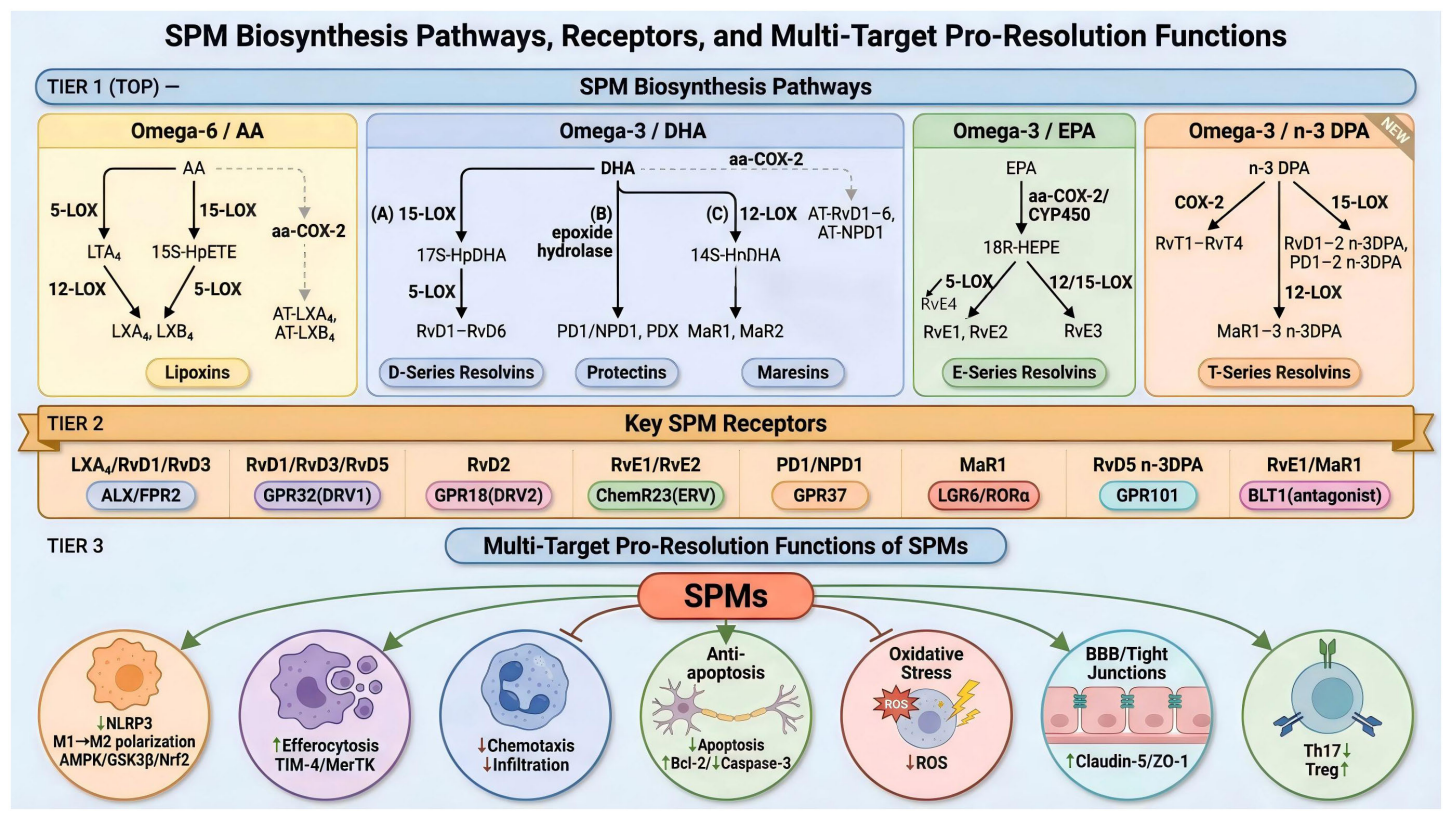
Schematic overview of SPM biosynthesis pathways, receptor interactions, and multi-target pro-resolution functions. This image systematically illustrates the generation of Specialized Pro-resolving Mediators (SPMs) from polyunsaturated fatty acid precursors, their specific receptor targets, and the mechanisms by which they actively resolve inflammation. The diagram is organized into three main sections (1): SPM Biosynthesis Pathways, detailing the enzymatic conversion of four major omega-3 and omega-6 fatty acids into distinct SPM families; (2) Key SPM Receptors, highlighting that SPMs do not directly kill pathogens but instead initiate programmed resolution by binding to specific G protein-coupled receptors (GPCRs) to transduce signals; and (3) Multi-Target Pro-Resolution Functions of SPMs, depicting how SPMs coordinate diverse immune cell behaviors to facilitate an orderly resolution of the inflammatory response. The central orange ellipse represents the core SPMs (including LXA4, RvD1, RvD2, MaR1, NPD1, and RvE1), radiating outwards to demonstrate their seven distinct biological functions in promoting inflammation resolution.

In terms of direct tissue protection, SPMs also exhibit multi-target advantages. They can act directly on the various cells that constitute the neurovascular unit to maintain the integrity of the BBB. For example, LXA_4_, through its ALX/FPR2 receptor expressed on endothelial cells, pericytes, and astrocyte end-feet, synergistically upregulates the expression of tight junction proteins (e.g., Claudin-5) to stabilize the BBB (62, 63). Furthermore, some SPMs have direct neuroprotective effects. NPD1 can act directly on neurons, protecting them from death due to oxidative stress or excitotoxicity by upregulating anti-apoptotic proteins like Bcl-2 and inhibiting the activation of key apoptotic executioner molecules like caspase-3 (64, 65). In summary, SPMs, through multi-target and multi-pathway synergistic actions, collectively form the core regulatory network for inflammation resolution and tissue repair (**Figure 2**).

Interestingly, recent evidence suggests that the endocannabinoid system may also contribute to pro-resolving responses. The endocannabinoid anandamide (AEA) has been shown to initiate pro-resolving pathways in macrophages by engaging both CB_2_ and GPR18 receptors, enhancing efferocytosis and modulating SPM biosynthesis (66). Notably, GPR18 is also a receptor for the SPM resolvin D2 (67), indicating direct crosstalk between these lipid mediator families. Furthermore, the phytocannabinoid cannabidiol (CBD) can induce a lipid mediator class switch by activating 15-lipoxygenase-1, promoting SPM formation while suppressing pro-inflammatory leukotrienes (68, 69). These findings illustrate that other immunomodulatory lipids may exert pro-resolving actions either by engaging SPM receptors or by promoting endogenous SPM biosynthesis, revealing a broader network of lipid mediators that orchestrate inflammation resolution.

## 4 Failed inflammation resolution: a core pathological mechanism of cerebral ischemia-reperfusion injury

For clarity, we operationally define “resolution dysfunction” in this review as a pathological state meeting at least two of the following three criteria: (1) a statistically significant reduction in plasma or tissue levels of key SPMs (e.g., LXA_4_, RvD1) relative to healthy controls or pre-injury baseline; (2) a persistently elevated pro-inflammatory/pro-resolving mediator ratio (e.g., LTB_4_/LXA_4_ or LTB_4_/RvD1) beyond the acute injury window (>72 hours post-ictus) (70); and (3) failure of the inflammatory microenvironment to transition toward a pro-reparative phenotype within the expected temporal window, evidenced by persistent M1 microglial predominance or impaired efferocytosis (71). We explicitly acknowledge that formal clinical thresholds for these criteria have not yet been validated in prospective interventional trials, and their standardization constitutes a critical research priority outlined in Section 7.2.

Although the body has evolved sophisticated inflammation resolution programs, this endogenous protective mechanism often fails under the severe pathological impact of cerebral ischemia-reperfusion. The central argument of this chapter is that “failed inflammation resolution” constitutes the key pathological link between acute neuroinflammation and chronic neurological damage. We will elaborate on this from two aspects: first, by exploring why the SPM biosynthesis pathways are impaired in the post-stroke brain microenvironment; and second, by analyzing how the absence of this “pro-resolving” signal leads to a series of detrimental consequences, ultimately causing the inflammatory response to persist and continuously damage brain tissue.

### 4.1 Impaired SPM biosynthesis in the post-stroke brain

The most direct evidence for the core hypothesis of “failed inflammation resolution” comes from the precise quantitative analysis of lipid mediators in injured tissues using liquid chromatography-tandem mass spectrometry (LC-MS/MS) lipidomics. Evidence from lipidomic studies in vascular ischemic disease models demonstrates that SPM levels (including RvD1 and LXA_4_) are significantly reduced and SPM/LTB_4_ ratios are markedly imbalanced in ischemic lesions (72). In the ischemic brain specifically, the failure of endogenous NPD1 biosynthesis to increase proportionally to the inflammatory stimulus is inferred from the observation that exogenous DHA/NPD1 administration consistently produces dose-dependent neuroprotection (73), suggesting a relative pro-resolving signal deficit rather than a documented absolute decrease. Direct LC-MS/MS quantification of SPM temporal dynamics specifically in ischemic penumbra vs. core tissue across different stroke models remains an important area for future investigation.

This production impairment is considered to be the result of multiple contributing factors. First, the explosive oxidative stress triggered by ischemia-reperfusion is a key inhibitory factor (74). The active sites of key enzymes in the SPM synthesis pathway, such as LOX and COX, often contain easily oxidizable iron ions (75). A large amount of ROS can directly attack these active sites, leading to conformational changes and loss of enzyme activity (76). A second contributing factor is substrate availability. Under conditions of intense oxidative stress, arachidonic acid and other PUFAs can undergo non-enzymatic free radical-catalyzed peroxidation, generating isoprostanes — structurally distinct, non-enzymatic oxidation products of arachidonic acid that are chemically different from enzymatic hydroperoxides (77). While isoprostanes themselves have limited direct pro-resolving activity, and some isoprostane species (e.g., 8-iso-PGF_2α_) have demonstrated vasoconstrictive and platelet-activating properties *in vitro (*78), whether this pathway meaningfully diverts substrate away from SPM biosynthesis *in vivo* remains to be directly demonstrated. We present this as a plausible mechanistic hypothesis consistent with the high oxidative burden observed in ischemia-reperfusion, pending experimental validation. Third, the efficient synthesis of SPMs often relies on close collaboration between different cell types. The microcirculatory disturbances, widespread activation and damage of vascular endothelial cells, and dysfunction of platelets caused by stroke severely disrupt this delicate intercellular collaborative microenvironment (e.g., the interaction between platelets and leukocytes is crucial for the transcellular synthesis of LXs), thereby further inhibiting SPM generation (79). Finally, many comorbidities common in stroke patients, such as diabetes and hypertension, further exacerbate the impairment of SPM synthesis. For example, in a high-glucose environment, the accumulation of advanced glycation end products (AGEs) can directly inhibit LOX enzyme activity, significantly reducing SPM production.

An additional mechanism contributing to impaired SPM biosynthesis involves the metabolic shunting of common intermediates. The unstable epoxide intermediate leukotriene A_4_ (LTA_4_), generated from arachidonic acid via the 5-lipoxygenase pathway, serves as a key branch point. Under physiological conditions, LTA_4_ can be either converted by LTA_4_ hydrolase into the potent pro-inflammatory chemoattractant leukotriene B_4_ (LTB_4_), or transcellularly metabolized by platelet-derived 12-lipoxygenase to generate pro-resolving lipoxin A_4_ (LXA_4_) and lipoxin B_4_ (LXB_4_) (37, 38). This diversion of LTA_4_ from the LTB_4_ pathway toward lipoxin biosynthesis represents a critical endogenous “switch” that governs the transition from inflammation to resolution. In the stroke pathological environment, oxidative stress-induced enzyme inactivation, disrupted platelet-leukocyte interactions, and insufficient substrate availability can impair this shunt, shifting the balance toward sustained LTB_4_ production and failed resolution, thereby perpetuating neuroinflammation and secondary tissue damage.

### 4.2 Deleterious consequences of failed resolution

The impairment of SPM production leads to a failure of the “braking system” of the inflammatory response, which in turn triggers a self-reinforcing pathological vicious cycle. First, due to the lack of potent promotion of phagocytosis by SPMs, macrophages and microglia cannot efficiently clear the large number of apoptotic neutrophils in the injured area. These uncleared apoptotic cells eventually undergo secondary necrosis, rupturing and releasing their stored DAMPs (e.g., HMGB1, histones) and proteolytic enzymes. This is tantamount to detonating new “bombs” in an already out-of-control fire, triggering a new round of inflammatory responses by activating receptors like TLRs, thus forming a vicious cycle of “clearance failure-recurrent inflammation” (14). Second, in a microenvironment lacking key pro-resolving signals like SPMs, persistent pro-inflammatory signals (e.g., IFN-γ) “lock” microglia/macrophages in a pro-inflammatory M1 phenotype by activating transcription factors like STAT1 (80). These chronic M1-type cells continuously secrete ROS, nitric oxide (NO), glutamate, and large amounts of pro-inflammatory cytokines, exerting a sustained toxic effect on surrounding neurons and oligodendrocytes. This is a key factor leading to secondary neuronal loss and demyelination (81). Finally, this persistent, non-resolving inflammatory state poses a serious obstacle to endogenous repair programs. It not only hinders the proliferation of neural stem cells and their differentiation into neurons by inhibiting crucial signaling pathways for neurogenesis, such as Wnt and Notch (82, 83), but also promotes the excessive activation and proliferation of astrocytes, forming a glial scar that inhibits axonal regeneration. It is noteworthy that some molecules that play a beneficial role in normal repair processes may show their duality in the context of dysregulated inflammation resolution. For example, TGF-β is a key anti-inflammatory factor in the acute phase, but its sustained high expression in a chronic inflammatory environment promotes the excessive secretion of chondroitin sulfate proteoglycans (CSPGs) and other extracellular matrix components by astrocytes that inhibit axon growth, ultimately forming a glial scar that physically and chemically obstructs the reconstruction of neural circuits (84). This profoundly reveals that successful tissue repair depends on the integrity and coordination of the entire pro-resolving program, rather than the isolated action of a single molecule.

## 5 Dysregulation of inflammation resolution in human stroke: clinical evidence and correlational analysis

Directly linking the phenomenon of “failed inflammation resolution” observed in preclinical models to the pathophysiological processes of human stroke is a critical step in validating its clinical relevance and advancing it as a therapeutic target. Although most mechanistic studies on SPMs are derived from animal models, a growing body of clinical research is providing direct or indirect evidence from multiple dimensions for the existence of the “failed resolution” hypothesis in human stroke patients. This chapter will systematically review this clinical evidence, which serves as a crucial “translational bridge.”

At the clinical level, the most direct evidence comes from lipidomic analysis of body fluid samples from acute ischemic stroke patients. Several studies analyzing patients’ peripheral blood or cerebrospinal fluid have found that, compared to healthy controls, the levels of various SPMs (e.g., LXA_4_, RvD1) are significantly reduced in stroke patients, while the levels of their pro-inflammatory counterparts—such as LTB_4_—are correspondingly elevated, leading to a severe imbalance in the “pro-resolving/pro-inflammatory mediator ratio” (70, 85). A recent meta-analysis integrating several such studies further confirmed the strong association between universally lower circulating SPM levels in stroke patients and poor prognosis. More importantly, some prospective cohort studies have linked this imbalance to specific clinical endpoints. For example, one study showed a significant negative correlation between the level of circulating SPMs at hospital admission and the patient’s functional outcome at 3 months (as measured by the modified Rankin Scale (mRS) score), meaning lower SPM levels predicted poorer functional recovery (70, 85). Furthermore, reduced SPM levels have also been associated with an increased incidence of post-stroke complications, such as cognitive impairment, depression, and other neurological complications (86, 87), suggesting that dysregulation of the inflammation resolution program may have systemic effects.

Genetics and epidemiological studies provide indirect but equally important support from different angles. The efficiency of SPM biosynthesis varies among individuals, partly depending on the genetic polymorphisms of the genes encoding their key synthesis enzymes (e.g., 5-LOX, 12-LOX, 15-LOX). Genome-wide association studies (GWAS) and candidate gene studies have linked certain single nucleotide polymorphisms (SNPs) in the ALOX5 gene (encoding 5-LOX) and the ALOX12 gene (encoding 12-LOX) to the vulnerability of carotid atherosclerotic plaques and the risk of stroke (88, 89). This suggests that an individual’s genetic background may preset the strength of their endogenous inflammation resolution capacity, thereby influencing their pathological response to ischemic injury. Meanwhile, numerous studies consistently show that a Mediterranean-style diet, rich in omega-3 PUFAs (DHA and EPA), is associated with a lower risk of stroke incidence (90, 91). Although this protective effect is multifactorial, one of its core biological bases is the provision of ample precursors for SPM synthesis. However, it is worth noting that the results of interventional clinical trials on whether direct supplementation with omega-3 PUFA preparations can effectively prevent stroke are not entirely consistent, which may be related to various factors such as study design, supplement dosage, and baseline omega-3 levels of the population (92, 93). But these negative findings do not undermine the SPM hypothesis—rather, they reinforce the core argument of this review. As discussed in Section 4.1, the post-stroke brain microenvironment is characterized by impaired endogenous SPM biosynthesis due to oxidative stress, enzyme dysfunction, and substrate competition. Therefore, simply providing more PUFA precursors may be insufficient to overcome this “biosynthetic bottleneck.” This reasoning supports the rationale for bypassing the dysfunctional enzymatic machinery by administering pre-formed, stable SPM analogs—a strategy distinct from precursor supplementation.

It is important to acknowledge that the clinical evidence summarized above, while supportive of the “failed resolution” hypothesis, possesses inherent limitations. Many of the cited human studies have focused on a limited panel of SPMs—most commonly RvD1 or LXA_4_—rather than conducting comprehensive lipid mediator profiling. Furthermore, some studies have relied on immunoassays (e.g., ELISA) for SPM quantification. However, as noted in the literature, ELISA-based measurements are prone to cross-reactivity and limited specificity, and are not widely accepted for precise quantification of structurally similar lipid mediators such as SPMs (38). Liquid chromatography-tandem mass spectrometry (LC-MS/MS) with authentic standards has become the gold standard for SPM identification and quantification due to its sensitivity and specificity (38, 67). Therefore, future studies employing comprehensive lipid mediator metabololipidomics via LC-MS/MS are urgently needed to validate the “failed resolution” hypothesis and to establish the translational potential of SPM-based therapies.

Beyond methodological considerations, a critical limitation of the current literature is the predominant reliance on preclinical animal models to establish mechanistic claims, whereas human data remain largely correlative in nature. While animal studies have provided valuable insights into the biosynthesis and function of SPMs, the translational gap between these models and human stroke pathophysiology limits our ability to infer causality between impaired resolution and long-term neurological deterioration. This imbalance underscores the need for well-designed human studies that can move beyond association toward mechanistic validation.

Furthermore, the interpretation of clinical evidence is complicated by the inherent heterogeneity of stroke populations. Critical variables such as age, sex, comorbidities (e.g., diabetes, obesity), and concomitant medications (e.g., statins) are known to influence lipid mediator biosynthesis and inflammatory responses (37, 38). However, most clinical studies have not adequately addressed these confounding factors, which may obscure the relationship between resolution dysfunction and neurological outcomes. Future investigations should stratify patients according to these variables and employ comprehensive lipid mediator profiling to better delineate the role of impaired resolution in human stroke pathophysiology.

In summary, while the existing clinical evidence collectively supports the presence of resolution dysfunction in human stroke, it is essential to recognize its correlational nature and methodological limitations. These caveats do not diminish the promise of pro-resolving therapies; rather, they highlight the critical steps—particularly direct human interventional trials with stable SPM analogs—that remain to be taken before clinical translation can be realized.

## 6 Targeting inflammation resolution: SPMs as a new strategy for stroke therapy

Based on the core hypothesis that “failed inflammation resolution” is a key link in post-stroke neurological damage, a logical therapeutic strategy emerges: if the endogenous pro-resolving program fails, then exogenously administering SPMs or their stable analogs can compensate for the deficit in pro-resolving signals, thereby actively controlling inflammation and guiding tissue repair. This represents a “pro-resolving” therapeutic philosophy, distinctly different from traditional “anti-inflammatory” strategies. Its goal is not simply to suppress inflammation but to restore the body’s own homeostatic regulatory capacity. This chapter will focus on reviewing the preclinical research evidence supporting this novel strategy and delve into its potential multi-target mechanisms of action.

### 6.1 Promising evidence from preclinical studies

Over the past decade, a large body of published preclinical research has provided a solid evidence base for the potential of SPMs as candidate drugs for stroke therapy. In various rodent models of stroke, including both transient (tMCAO) and permanent (pMCAO) middle cerebral artery occlusion models, the exogenous administration of SPMs or their more chemically stable synthetic analogs has consistently shown significant neuroprotective effects. The D-series resolvins (RvDs) are one of the most extensively studied families. A representative study in a mouse tMCAO model showed thatRvD1 treatment significantly enhanced the protective effects on the ipsilesional penumbra, as evidenced by an increased expression of microglial and astrocyte genes associated with anti-inflammatory signaling, synaptic circuitry protection, and neuronal cell survival (94). Another study on RvD2 in a rat tMCAO model, where 10 ng/kg of RvD2 was administered intraperitoneally at 1 and 6 hours post-reperfusion, also observed a reduction in brain edema and neuronal apoptosis, with the mechanism being closely nNOS/eNOS-mediated anti-apoptotic effects, and upregulated tight junction protein ZO (95).

As an SPM specifically expressed in the nervous system, the neuroprotective effect of Neuroprotectin D1 (NPD1) has also been fully validated. *In vitro*, NPD1 can effectively protect neurons from glutamate excitotoxicity and oxidative stress damage by upregulating anti-apoptotic proteins like Bcl-2 (57). In a MCAO model, NPD1 improved behavior, reduced lesion volumes, protected ischemic penumbra, increased NeuN, GFAP, SMI-71-positive cells and vessels, axonal regeneration in the penumbra, and attenuated blood-brain barrier (BBB) after MCAO (73).

Other SPM family members have also shown unique therapeutic advantages. Lipoxin A_4_ (LXA_4_) and its stable analogs have demonstrated outstanding performance in maintaining the integrity of the BBB. A study in a rat MCAO model showed tha Brain edema and Evans Blue leakage were significantly reduced after stroke in the LXA (4)ME group and were associated with reduced brain infarct volumes. MMP-9 activity and expression were inhibited by LXA (4)ME after stroke (96). Maresin 1 (MaR1) has shown powerful pro-reparative potential. Studies have shown that in a mouse tMCAO model, intracerebroventricular injection of 1 ng of MaR1 24 hours after reperfusion not only protected against cerebral IRR by reducing infarct volume but also effectively improved neurological deficits, and suppressing pro-inflammatory responses via inhibition of NF-κB p65 activation and nuclear translocation, thereby synergistically promoting neurogenesis and angiogenesis (97). These studies collectively paint a broad prospect for SPMs as multi-target, multi-potent therapeutic drugs for stroke (**Table 1**).

**Table 1:** Summary of representative studies on the therapeutic effects of key SPMs in preclinical stroke models.

SPM type	Animal model	Dosing regimen	Key findings (original key conclusion)	Reference
Resolvin D1 (RvD1)	Rat MCAO (6h)	RvD1 (111, 222, or 333 µg/kg), IV, 1h post-reperfusion	RvD1 treatment significantly enhanced the protective effects on the ipsilesional penumbra, as evidenced by an increased expression of microglial and astrocyte genes associated with anti-inflammatory signaling, synaptic circuitry protection, and neuronal cell survival.	(94)
Resolvin D2 (RvD2)	Rat tMCAO (120min)	25, 50, 100 µg/kg, i.p.	RvD2 bound to GPR18 activates ERK1/2 phosphorylation, enhanced nNOS/eNOS-mediated anti-apoptotic effects, and upregulates tight junction protein ZO-1, thereby preserving BBB integrity and mitigating early brain injury post-MCAO/R.	(95)
Neuroprotectin D1 (NPD1)	Rat tMCAO (120min)	5 μg/per rat, i.p.,	NPD1 improved behavior, reduced lesion volumes, protected ischemic penumbra, increased NeuN, GFAP, SMI-71-positive cells and vessels, axonal regeneration in the penumbra, and attenuated blood-brain barrier (BBB) after MCAO.	(73)
Lipoxin A_4_ (LXA_4_)	Rat MCAO (120min)	1 μg/kg, i.v., 24h post-reperfusion	Brain edema and Evans Blue leakage were significantly reduced after stroke in the LXA (4)ME group and were associated with reduced brain infarct volumes. MMP-9 activity and expression were inhibited by LXA (4)ME after stroke.	(96)
Maresin 1 (MaR1)	Mouse tMCAO (60min)	1 ng/mouse, i.c.v., 24h post-reperfusion	MaR1 significantly protected against cerebral IRR by reducing infarct volume, improved neurological deficits, and suppressing pro-inflammatory responses via inhibition of NF-κB p65 activation and nuclear translocation.	(97)

### 6.2 Mechanistic insights

The powerful neuroprotective effects of SPMs stem from the synergistic effects produced by the activation of multiple downstream signaling pathways after binding to their respective specific receptors. Their mechanisms of action are multi-dimensional and multi-targeted. First, at the core of inflammation inhibition, SPM molecules like RvD1 have been confirmed to directly inhibit the assembly and activation of the NLRP3 inflammasome within microglia. The specific mechanism may involve inhibiting the ubiquitination of NLRP3 or interfering with its binding to key adaptor proteins like NEK7, thereby effectively blocking the activation of caspase-1 and the subsequent maturation and release of key pro-inflammatory cytokines like IL-1β and IL-18 (98). Second, SPMs are key reprogrammers of immune cell function. Molecules like RvD1 and MaR1 can “push” microglia/macrophages from a pro-inflammatory M1 phenotype to a pro-reparative M2 phenotype by activating the AMPK signaling pathway, which in turn phosphorylates and inhibits glycogen synthase kinase 3β (GSK3β), ultimately promoting Nrf2 nuclear translocation and upregulating antioxidant gene expression (99, 100). Additionally, SPMs can downregulate the expression of M1-related genes by inhibiting the phosphorylation of STAT1 (101). Third, SPMs also play an important role in maintaining the integrity of the neurovascular unit. For example, LXA_4_, through its ALX/FPR2 receptor expressed on endothelial cells, pericytes, and astrocyte end-feet, synergistically enhances intercellular connections to stabilize the BBB (102). Some studies also suggest that SPMs can exert protective effects by regulating autophagy, for instance, by promoting the autophagic clearance of damaged mitochondria (mitophagy), thereby reducing oxidative stress and apoptosis (103, 104). Finally, some SPMs also have direct neuroprotective effects. NPD1 can act directly on neurons, protecting them from death by upregulating anti-apoptotic proteins like Bcl-2 and inhibiting the activation of key apoptotic executioner molecules like caspase-3 (65). These multi-target, multi-pathway synergistic actions collectively form the molecular basis for the powerful neuroprotective and pro-reparative functions of SPMs.

## 7 Challenges and future perspectives

### 7.1 Translational challenges

Despite the encouraging results from preclinical studies, successfully translating SPM therapy from the laboratory to clinical application still requires overcoming a series of significant scientific and technical challenges. First, in terms of pharmacokinetics and drug delivery, natural SPMs, as lipid molecules, have extremely short *in-vivo* half-lives (usually only a few minutes) and are easily and rapidly metabolized and inactivated, which severely limits their druggability (105). To address this challenge, current research strategies are mainly focused on two directions: one is to develop chemically modified, metabolically stable synthetic analogs that are insensitive to metabolic enzymes (106); the other is to use advanced drug delivery systems, such as liposomes, polymer nanoparticles, exosomes, or hydrogels, to encapsulate SPMs (107). This can achieve sustained release, prolong circulation time, and potentially enable targeted delivery to the ischemic brain tissue. Furthermore, exploring non-invasive administration routes that can bypass the BBB, such as intranasal delivery, has shown preliminary success in some preclinical models, but its delivery efficiency in humans still needs to be validated (108). Second, determining the therapeutic window is another key challenge. The optimal timing for administering SPMs is still unclear. Could the use of potent pro-resolving drugs too early (e.g., in the very early stages of the inflammatory response) interfere with the necessary debris clearance function of early inflammation? Most animal experiments show that administration within a few hours after reperfusion is most effective, but this is difficult to achieve in clinical practice for most stroke patients (67, 109). Therefore, it is crucial to explore and determine a wider, clinically feasible effective therapeutic window. Moreover, the optimal therapeutic window may also be influenced by various factors such as stroke subtype, patient age, and comorbidities (110, 111). Finally, the optimization of treatment regimens, including the best dosage and drug selection, also needs to be addressed. Determining an optimal dose that can effectively initiate the pro-resolving program without causing off-target effects or receptor desensitization due to excessively high doses requires extensive dose-response relationship studies. Furthermore, considering the varying pathological features at different time points after a stroke, single-SPM molecule therapy may not be the optimal solution. In the future, developing “cocktail” combination therapies containing multiple different SPM molecules that can be administered sequentially (“sequential therapy”) may be a strategy that better aligns with the dynamic changes in pathophysiology (94, 112). At the same time, although SPMs, as endogenous molecules, are theoretically highly safe, they must undergo rigorous preclinical toxicology assessments before entering clinical trials as drugs to rule out potential effects on other physiological processes such as coagulation and blood pressure. Their complex stereochemical structures also pose significant technical challenges for large-scale, high-purity chemical synthesis that complies with Good Manufacturing Practice (GMP) standards.

### 7.2 Key unanswered questions

To steadily advance this promising new therapeutic paradigm toward clinical application, the entire field must focus on answering the following interrelated and progressively deeper key scientific questions.

First is the fundamental dynamic mapping problem: What are the spatiotemporal dynamic expression profiles of SPMs, their synthesis enzymes, and receptors in the ischemic core, penumbra, and remote areas of the human stroke brain? Current data are mainly from peripheral blood or rodent models. Using emerging spatially resolved multi-omics technologies (such as spatial transcriptomics, spatial lipidomics) and single-cell sequencing technologies (such as single-cell RNA sequencing (scRNA-seq), single-cell Assay for Transposase-Accessible Chromatin using sequencing (scATAC-seq)) to directly map a high-resolution “inflammation resolution atlas” of the human brain is crucial for precisely identifying key cell subpopulations, molecular targets, and therapeutic time windows (113–115).

Second is the core causality problem: Is the clinically observed decrease in SPM levels merely a “consequence” of the stroke pathological process, or is it an intervenable “cause” of poor outcomes? (116) To answer this question, in addition to validation in more complex animal models with common clinical comorbidities like hypertension and diabetes, it is necessary to combine more advanced epidemiological methods such as Mendelian Randomization (MR) (117, 118). Using genetic variations in the key enzymes of the SPM synthesis pathway as instrumental variables can more reliably infer the causal relationship between them and stroke prognosis (116).

Third is the technical precision targeting problem: Can we develop “smart” SPM prodrugs or delivery systems that can be activated by the specific pathological microenvironment after a stroke (such as low pH, high levels of ROS, or high expression of specific enzymes)? For example, a prodrug could be designed by linking an SPM molecule to a nanocarrier via a ROS-sensitive chemical bond (such as a thioether bond). When it reaches the ischemic area with high ROS levels, the chemical bond breaks, achieving site-specific drug release. This “pathology-responsive” delivery strategy holds the promise of solving the core problems of poor stability of natural SPMs and potential off-target effects of systemic administration, and it is the technical key to achieving precise “pro-resolution” therapy (107, 119).

Finally, there is the biomarker problem directly facing clinical application. We propose that a clinically actionable “Resolution Index (RI)” should be a composite scoring system incorporating the following candidate components: (1) plasma levels of RvD1 and LXA_4_ quantified by LC-MS/MS (gold standard method); (2) the LXA_4_/LTB_4_ and RvD1/LTB_4_ ratios as indicators of the pro-resolving/pro-inflammatory balance; (3) monocyte surface expression of MerTK as a cellular marker of efferocytic capacity; and (4) ALOX5/ALOX12 SNP status as a genetic correction factor for inter-individual biosynthetic variability. Importantly, the feasibility of this approach is grounded in existing clinical data: a prospective study of acute ischemic stroke patients undergoing endovascular thrombectomy demonstrated that post-procedural LXA_4_/LTB_4_ ratio and RvD1/LTB_4_ ratio were independent predictors of early neurological deterioration and unfavorable 90-day functional outcome (70, 120), providing the closest existing clinical approximation of a resolution index. We explicitly acknowledge that no standardized RI threshold currently exists, and its formal validation will require: (i) large prospective cohort studies to establish normative ranges and outcome-linked cut-off values; (ii) accessible, high-throughput LC-MS/MS platforms; and (iii) Mendelian Randomization studies to establish causality between impaired resolution and stroke outcomes. Until validated, the RI serves as a hypothesis-generating framework to guide patient stratification in future SPM interventional trials.

## 8 Conclusion

The traditional view of post-stroke neuroinflammation as a purely destructive process that needs to be completely suppressed is facing dual challenges from both basic biology and clinical translational practice. The paradigm shift from “passive anti-inflammation” to “active pro-resolution” proposed in this review provides a new theoretical framework for understanding and intervening in this complex pathological process. By integrating multi-dimensional evidence from molecular mechanisms, cellular functions, to clinical observations, this review systematically argues that “failed inflammation resolution” is the core mechanism linking acute cerebral ischemia-reperfusion injury to chronic neuropathological deterioration. The production impairment and functional dysregulation of Specialized Pro-resolving Mediators (SPMs), as the key executors of this endogenous program, in the stroke pathological environment provide a profound and logically consistent explanation for the persistence of the disease and the failure of repair. Although translating SPM therapy into clinical practice still requires overcoming numerous challenges in drug delivery, therapeutic windows, and precision targeting, targeting and restoring the brain’s own inflammation resolution function represents a biologically rational direction for next-generation stroke therapy development. However, the path to clinical application remains long, and its successful navigation requires not only solutions to drug delivery and safety challenges but also rigorous validation in direct human interventional trials. One such approach involves strategies that amplify endogenous SPM pathways by targeting their interplay with endocannabinoid systems offer promising alternatives to direct SPM administration. This is not just an exploration of a new class of drugs, but a profound practice of a disruptive therapeutic philosophy aimed at “working with the body, not against it,” with the ultimate goal of restoring tissue homeostasis.
